# The Spirit Rapper

**Published:** 1854-06

**Authors:** 


### THE SPIRIT RAPPER.

(A TRUE STORY.)

WRITTEN BY A CLERGYMAN,

*Illustrating the evil influence of Over-eating.*

SOME few years ago, when the Spirit Rapping manifestation seemed to have aroused the public mind, and the well-known excitement which then followed pervaded every house to more or less extent, it fell to my lot to become personally acquainted with a young theological student, who narrated to me the following graphic occurrence:—

"I was intensely excited upon hearing of the visible, audible appearance, and manifestations of spirits, from the hitherto unseen world. I took it for granted at once that such communications were sure—beyond all doubt—folly to express a doubt. I felt almost afraid to surmise that it were possible to be true or false. In short, it was reduced to a certainty that the spirits existed, and that I, myself, should become a Medium forthwith.

"I thought about the subject all day. I dreamed about it all night. With all who would talk, I talked; and wondered that anybody should not feel enraptured, as I felt. Walking along, riding, sitting, reading,—spirits, spirits! I saw, nor thought of any thing than spirits. It may seem strange to the reader that I should have been so enthusiastic at an event that certainly was not demonstrated, and, by almost every one, very much doubted. But I was young; and, I was 'green,' you may add. Certainly, I was very credulous. Had no experience in the world, except with good men, consequently never knew the bad part of it. At all events I thought this—that the spirits, good or bad, could not get access to us without Divine permission, therefore, their communion with us was intended to accomplish some good object. I never doubted that the spirits did not hold intercourse in the 'rapping' way.

"One night I retired to rest, after having eaten rather heartily, and felt as though I had rather gorged myself too much, for the good things were plenty and abundant around me. Calmly reposing under the shades of the night until about ten o'clock, I thought I heard a singular sound in the north corner of the room. It was as dark as any night I ever saw. To look in that direction with the view of seeing any thing was preposterous—to lay still and do nothing seemed to me to be equally so. It occurred for a moment that to *hallo right out* would be the only alternative, but then it looked rather silly to alarm a neighborhood unless there were some real apprehensions of murder, or thieves in the apartment. These were the only three courses I could take, and which were the best in my case of undue excitement the reader may judge. I thought, however, I could but *lie still*, as it was too dark for assassins to see the hiding corner I was in, and too well wrapped up to be seriously molested by the spirit tribe. Here I was determined to await my fate. The noise was such that it could not be disputed that something did it. It was an unearthly, singular noise. I had never heard such before in my life, and what made it most singular was that,—the time was midnight, the very hour when spirits come. I found that it was folly to quiet the rapping by saying nothing. I felt that no thief could be the perpetrator, for he would not have tantalized me a whole hour. I knew it was not a cat, for the noise was the same all the time—that at intervals. By-and-bye I aroused myself from the seeming lethargy I was in; I determined to speak—to say something!—I felt that thing was a spirit; sure—for how could it be anything else? I thought for a moment, now 'spirits will not talk unless you speak first,'—well, here it goes—hem!

"'Who are you?'

"No reply.

"(Louder tone)—'Who are you?'

"No reply.

"'My dear deceased sister, is that you?'

"'Flip, flop! flip, flop!!!'

"'Ah! it is you.'

"'Flip, flop! flip, flop!!!'

" 'Well, what do you want ?'

" 'Flip!'

" 'What will—'

" 'Flip, flip!!'

" 'What will you ha—'

" 'Flop, flip! flip, flop!!'

" 'Have ?'—

" 'Dear sister, communicate your wish to a distracted—'

" 'Flip, flop! flip, flop!!'

" (The spirit treats me with contempt)—'Are you the spirit of grandfather ?'

" No reply.

" 'Are you the spirit of grandmother ?'

" (Taking courage.)—'What thundering spirit are you then ? You are none of my acquaintance. Are you the evil one ?'

" 'Flip, flop! flip, flop!! flip, flop!!!'

" 'I say, are you the evil one,—and what do you want here ?'

" 'Get thee behind me, Satan.'

" 'Sp'l, d'l'ash, blup, blup, flip.'

" 'What, under heaven, are you ? A, B, C, D E—?'

" 'Flip.'

" 'A ?'

" 'Ah, A is the letter. What next ? B, C, D, E, F, G, H, I, J, K, L, M, N, O, P, Q, R, S—?'

" 'Flop,—!!!'

" 'S is the letter, but then it rapped at S twice, that means double S, A,SS!—Pshaw! nonsense. You are one yourself. I say, I believe you are the evil one without any intelligence, so go away.'

" At this time the sweat began to roll down the face in profusion, and there was left nothing more to do than to raise the alarm, for if it was the devil his eternal 'flip, flop,' would not cease by my request. While these thoughts were cogitating there was an incessant 'flipping and flapping,' which very plainly showed that nothing I had done had the least tendency to quell the disorder.

" 'Flip, flop! flip, flop!'

" 'Ting—ting—ting—ting!'

" 'What ? Five o'clock in the morning!!! By the stars—

it is five o'clock in the morning, and my head immersed in this blanket has kept out day-light this hour! Leaping out of bed—in rushed father and mother, one with the shovel, the other with the tongs, and Julia with the broom.

"'What on earth is the matter?' all cried out with one breath.

"Matter! enough is the matter. The "old boy" has been here last night, and, and frightened—'

"'Flip, flop!'

"'There! Hear him! He has been doing his wings that way all night, and for the life of me I thought I was gone more than once. What now! Didn't you hear the rapping yourself?'

"'Yes; but it is nothing more than a poor little mouse, half drowned in the wash basin, and there he is now, with his head only out of the water!'

"Readers, it was the fact!—*the Rochester Spirit and the evil one was nothing more than a drowning mouse!*"

Moral—Avoid hearty suppers. Q. E. D.

